# Complications associated with talectomy in paediatric patients: a comparative retrospective study of two surgical techniques

**DOI:** 10.1186/s12891-021-04309-2

**Published:** 2021-05-07

**Authors:** Thamer S. Alhussainan, Omar A. Al-Mohrej, Abdullah Y. Almarshad, William J. Wade

**Affiliations:** 1 Department of Orthopedics King Faisal specialist hospital and research centre, Riyadh, Saudi Arabia; 2 Division of Orthopaedics, Department of Surgery, McMaster University, Hamilton, ON Canada; 3 Section of Orthopedic Surgery, Department of Surgery, King Abdullah Bin Abdulaziz University Hospital, Princess Nourah Bint Abdul Rahman University, Riyadh, Saudi Arabia

**Keywords:** talectomy, pediatric, complications, recurrence, foot deformities

## Abstract

**Background:**

Studies describing the surgical approaches utilized for talectomy and their associated complications are scarce. We aimed to compare the surgical techniques and associated procedures from two groups of patients who underwent talectomy using two approaches. The main purpose of this study was to describe the complications and recurrence rates associated with each technique.

**Methods:**

Between January 2004 and December 2019, 62 talectomies were performed in 48 pediatric patients with different pathologies. All patient data were reviewed retrospectively, and data of 31 patients were included in the study. The patients were divided into two groups based on the surgical technique used, and the baseline characteristics, along with the post-operative findings, and the intervention types in relation to complications were analyzed.

**Results:**

In the terms of hindfoot varus, midfoot adductus, forefoot supination, and dorsal bunions, the prevalence of these deformities was higher in group (A). Group (B) patients tolerated braces (88.9 %) better than group (A) patients (84.0 %). More adjunct procedures were required in group (A) than group (B) Furthermore, the frequency and types of complications, as well as the need for further surgeries were also higher in group (A). There was a higher rate of recurrence in group A than group B.

**Conclusions:**

Talectomy is an effective procedure for the treatment of persistent foot deformities despite associated complications. Surgical details and addressing associated deformities with adjunct surgical interventions should be considered.

## Background

Talectomy is rare in modern orthopedic surgery, although it has been performed for many years. It was first performed to treat deformities in the talus caused by tuberculosis in an adult [1]. Whitman described employment of talectomy for a variety of paralytic deformities [2, 3].

In 1971, Menelaus described that talectomy was useful in the management of the rigid talipes equinovarus [4]. Since then, talectomy has been consistently used in the field of pediatric orthopedics, mainly for persistent rigid foot deformities, with reasonable results [5–7].

Since talectomy leads to inadequate bony support in the medial column of the foot, subsequent hindfoot equinovarus, midfoot adductus, and forefoot supination deformities have been observed [5, 8]. Thus, many additional procedures, such as navicular excision and calcaneocuboid fusion have been associated with the initial procedure [9, 10].

Additional procedures aim to reduce the rate of recurrence, which has been noted to occur between two and six years after talectomy [8]. Residual deformities have been reported as one of the most common complications resulting from incomplete removal of the talus, improper positioning of the calcaneus in the ankle mortise, or malreduction of the midfoot on the newly built hindfoot [4]. Therefore, attention to surgical detail may prevent both recurrence and other deformities [11].

To our knowledge, there are few studies describing the different surgical approaches utilized in talectomy, and their impact on associated complications. In this study, we aimed to compare two groups of children with different disorders who underwent talectomy by two different approaches due to persistent severe foot deformities. Our main objective was to describe the complications associated with each technique, as well as the rate of recurrence.

## Methods

### Study Setting

Between January 2004 and December 2019, we carried out 62 talectomies in 48 pediatric patients with different pathologies at the King Faisal Specialist Hospital and Research Centre (KFSH&RC), Riyadh, Saudi Arabia [12]. The pediatric orthopedic clinic in KFSH&RC is one of the few major facilities specialized in pediatric orthopedics in Saudi Arabia and receives referrals from all over the country. The clinic has three main branches; the brachial plexus and upper limb conditions clinic [13], the spine clinic [14], and the lower limb clinic [15]. The lower limb unit launched the National Hip Dysplasia Program (NHDP) as an outreach initiative to diagnose and surgically treat neglected developmental dysplasia of hip (DDH) patients at walking age in local hospitals, in order to reduce the surgical waiting time and the cost of travel and increase the awareness of the local hospitals about DDH diagnostic and therapeutic implications [16]. In 2019, 4509 patients were seen in the lower limb clinic/NHDP and 507 surgeries of different pathologies were performed.

### Surgical indications

The indication for the surgery was severely rigid or relapsed talipes equinovarus in neuromuscular or syndromic patients who had no response to serial casting or soft tissue release. The two main diagnoses were arthrogryposis and myelomeningocele. Other diagnoses, such as Edward syndrome, grebe chondrodysplasia, and cerebral palsy (CP) were seen as well.

### Surgical techniques

Talectomies were performed mainly as salvage procedures but in some cases were used as primary procedures mainly in neglected clubfeet associated with either spina bifida or arthrogryposis. A trial of Ponseti casting was usually performed in the neglected non-operative cases but if it failed, the surgical correction typically started with posterior soft-tissue release (STR). If the STR was not sufficient to correct the hind foot deformity, talectomy was chosen as the primary procedure. Conversely, if a child with spina bifida or arthrogryposis also presented with failed or severely relapsed congenital talipes equinovarus (CTEV) after STR procedures, talectomy was carried out without posterior STR.

Initially, talectomy began in our center utilizing a modified skin incision that was medial to the one described by Menelaus [4]; this approach helped to isolate the dorsal neurovascular bundle. This incision started anterior to the medial malleolus and ended distal to the level of the midfoot between the first and second metatarsal. This medial incision makes exposure of the subtalar joint very difficult, which often results in failure to remove the talus as one piece; thus, the talus is mostly removed in fragments. After excision of the talus, the Achilles tendon was resected, and the posterior ankle capsule and anterior deltoid ligament that holds the navicular in the adducted position were released.

Reduction of the calcaneum was then attempted, and if the calcaneum was not set in the mortise properly, release of the anterior syndesmotic ligament and resection of the tip of the lateral malleolus was carried out to achieve the proper contact between the calcaneum and tibial pilon. The proper position of the calcaneum should include 5° of the valgus, sufficient posterior translation, and 20–40° of calcaneal pitch angle, which were monitored with intraoperative images. If proper calcaneal reduction was achieved, it was fixed with one or two retrograde calcaneotibial K-wires.

The next step was the reduction of the midfoot to the reduced and stabilized hindfoot. Initially, we were very conservative when adding midfoot bony procedures to achieve midfoot full correction due to concerns about the reduction in foot size. Residual mild midfoot adduction equivalent to a Pirani Score [17] of 0.5 for the lateral foot border was accepted and not considered an indication for midfoot bony procures. Occasionally, an additional midfoot stabilizing K-wire was added to hold the midfoot in the best achieved position. This initial group of patients receiving the talectomy procedures utilizing the medial modified approach and accepted residual adduction were labeled as group A.

The surgical approach of talectomy changed at the end of 2014 to the initially classically described incision. This incision starts over the anterolateral aspect of the ankle joint, just anterior to the lateral malleolus, passes through the sinus tarsi, and ends over the prominently subluxated talar head, which is at the level of the 4th intermetatarsal space. Using this approach, the talus was excised as one piece in all cases in this subgroup. The accepted position was a straight lateral foot border (Pirani score of 0 for the lateral border) dictating a lower threshold to perform midfoot bony procedures, mainly calcaneocuboid fusion [10]. This group of patients was labeled as group B.

In both subgroups, above knee casts were applied at the end of the procedure to maintain the position of the foot.

### Post-operative protocol

The postoperative protocol was the same in both groups. Continuous elevation of the limb was maintained for the first postoperative day. Patients remained in the hospital for one day and received medication for pain management (acetaminophen IV 15 mg/kg q6hr and morphine IV 0.05-1 mg/kg q4hr PRN for pain) and three doses of intravenous antibiotics (cefazolin IV 25 mg/kg q8hr).

Laboratory investigations were also performed for all patients on the first postoperative day. Antibiotics were not prescribed after discharge, but patients were given oral pain control medications (acetaminophen oral syrup 15 mg/kg q6hr).

Clinical follow-ups occurred 2 weeks postoperatively for suture removal, wound inspection, and one cast change. The cast and K-wires were removed at 6 weeks postoperatively.

The patients remained in custom-made ankle foot orthoses (AFO) based on measurements taken at the first postoperative visit. The AFO were gradually removed over the coming year. Follow-up appointments also occurred at 6 weeks, 3 months, 6 months, and then annually.

A physiotherapy (PT) program was initiated by a PT specialist for all patients post-cast removal. Educational materials, such as brochures, booklets, and videos, were given to the parents to educate them on how to do passive range of motion (ROM) exercises at home.

### Statistical analyses

Descriptive measures, such as frequencies, percentages, means, and standard deviations were used to describe quantitative and categorical data. Statistical analyses were conducted using Statistical Package for the Social Sciences (SPSS) (IBM Corp. Released 2016. IBM SPSS Statistics for Mac, Version 24.0. Armonk, NY: IBM Corp).

## Results

**Fig. 1:**
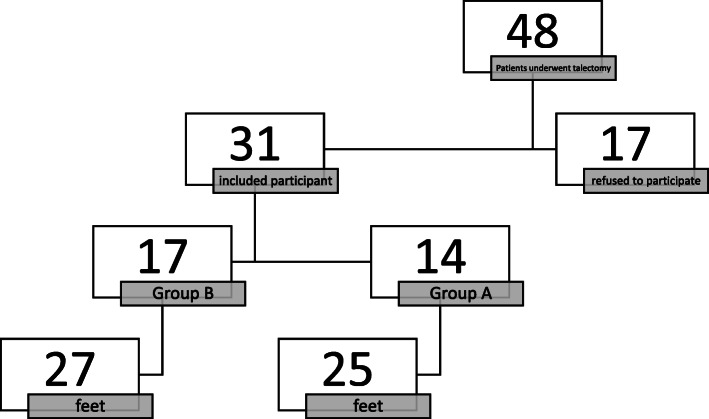
Flow diagram of the study participants

 The parents or legal guardians of 31 patients provided informed consent for the inclusion of their children in this study. The remaining 17 patients were excluded because of the lack of parental consent. So, of the eligible patients, 64.5 % were included (Fig. 1).

The mean follow-up time was 8.9 ± 3.5 years and 4.8 ± 3.2 years for group A and group B, respectively. Myelomeningocele was the predominant diagnosis in group A (48.0 %), whereas arthrogryposis and myelomeningocele were the most common diagnoses in group B (40.7 %). Talectomy was mainly performed as a primary procedure in both groups. Table 1 summarizes the baseline characteristics of the two groups.

**Table 1:** Baseline characteristics of the two groups

Variable	Categories	Patient Group			
		Group A *N* = 25		Group B *N* = 27	
		N	%	N	%
Age^a^ in years	Mean ± SD	6.0 ± 2.0		5.0 ± 3.0	
Mean Follow-up in years	Mean ± SD	8.9 ± 3.5		4.8 ± 3.2	
Primary diagnosis	Arthrogryposis	8	32.0	11	40.7
	Myelomeningocele	12	48.0	11	40.7
	Others	5	20.0	5	18.5
Type of talectomy	Primary procedure	20	80.0	21	77.8
	Salvage procedure	5	20.0	6	22.2
Side	Unilateral	4	16.0	5	18.5
	Bilateral	21	84.0	22	81.5

^a^ At the time of surgery

In the terms of hindfoot varus, midfoot adductus, forefoot supination, and dorsal bunions, the prevalence of these deformities was higher in group A. Group B patients tolerated braces (88.9 %) better than group A patients (84.0 %). The post-operative findings are presented in Table 2.

**Table 2:** The post-operative clinical findings among the two groups

Variable	Patient Group			
	Group A *N* = 25		Group B *N* = 27	
	N	%	N	%
Hindfoot varus	4	16.0	3	11.1
Midfoot adductus	8	29.6	5	20.0
Forefoot supination	6	24.0	6	22.2
Dorsal bunion	7	25.9	6	24.0
Tolerating braces	21	84.0	24	88.9

More adjunct procedures were required in group A than group B. Furthermore, the frequency and types of complications, as well as the need for further surgeries were also higher in group (A) There was a higher rate of recurrence in group A than group (B) The intervention types and associated complications are summarized in Table 3.

**Table 3:** The intervention types in relation to complications

Variable	Patient Group			
	Group A		Group B	
	*N* = 25		*N* = 27	
	N	%	N	%
Adjunct procedures^a^	1.9 ± 1.1		3.9 ± 2.5	
Complications	10	40	2	7.4
Recurrence	10	40	1	3.7
Vascular injury	0	0	1	3.7
Requiring further surgery	7	28	2	7.4
Time to reoperation^a^	2.1 ± 1.3		2.4 ± 3.6	

^a^ expressed as mean ± SD

## Discussion

Pediatric orthopedic literature includes a variety of surgical options that are used to treat persistent, severe foot deformities. Talectomy is included in these procedures, but should be reserved as a salvage procedure in many cases [18]. Our study covered 52 talectomies (31 patients) performed over a 15-year period, in which two different surgical methods were used. Earlier, we published the outcome of those patients with no surgical details [12]. There were substantial deficiencies in the surgical details. These differences were analyzed, in this study, to see the effect of different surgical approaches on the final outcomes.

Patients’ and their caregivers’ satisfaction was a predictor of successful outcomes regarding foot and ankle surgeries as per Al-Mohrej et al. [19]. The short-, mid-, and long-term talectomy outcomes were reported to be good [5–7]. However, the literature does not provide a comprehensive overview because most studies grouped patients, who underwent talectomy by different approaches, together. Additionally, studies describing the complications associated with talectomy are sparse [12].

Talectomy has been described both as a primary procedure and as a salvage procedure after recurrent injuries [7, 20]. Many studies suggested that talectomy should be the primary procedure for all persistent rigid foot deformities [21], while another reported a satisfactory rate of only 50 % for primary talectomies [22]. In this series, talectomy was mainly used as a primary procedure in all patients. This was necessary because of the late presentation of the patients. Ponseti method and simple tissue release would have been ineffective in our patient cohort as some patients had already undergone such procedures in prior surgeries. However, it is hypothesized that the results and complications observed in this study were related to the patients’ primary diagnosis and intraoperative attention to surgical details.

The complication rate for both groups was approximately 23.1 %. Nevertheless, the rates of hindfoot varus, midfoot adductus, forefoot supination, dorsal bunions, and recurrence were higher in group A. It could be argued that some complications were related to the mean follow-up time of group A (8.9 ± 3.5 years) compared to group B (4.8 ± 3.2 years). It is commonly thought in the field that recurrence generally results from technical issues during surgery, such as incomplete removal of the talus, incorrect positioning of the calcaneus in the ankle mortise, and performing the talectomy without additional procedures [6, 10, 18, 23]. In our study, Most of the revision cases were done 2 to 3 years postoperatively. Therefore, the difference in follow-up periods between the groups is not meaningful. Nonetheless, the main reasons for the recurrence rate of group A (40 %) versus group B (3.80 %) were incomplete removal of the talus and the talectomy was performed without additional procedures in many cases when necessary.

We believe that proper exposure of the talus, especially the subtalar joint, is key for complete talar resection, which was supported by changing the surgical approach to that used in group A. Many candidates for talectomy have marked external tibial torsion [24], which makes the skin incision more medial than it should be. A critical step during surgical preparation is to mark the lateral malleolus, the sinus tarsi, and the prominent head of talus. Starting a skin incision just anterior to the lateral malleolus, passing through the sinus tarsi, and ending distal to the prominent talar head provides sufficient exposure of the soft tissue attached to the talus, including the tough subtalar interosseous ligament. Increasing the deformity while manipulating the talus with a sharp towel clamp after releasing the attached soft tissues, permits complete talar removal with minimal damage to the calcaneal and distal tibial cartilage.

Contrastingly, vascular compromise, which was seen in one patient in group B, is thought to be related to the surgical approach used because the medial neurovascular bundle is not isolated and protected. We hypothesize that this complication was not a true vascular injury but was related to stretching of surrounding soft tissue because the intraoperative forefoot position in this subgroup was corrected more in an attempt to limit the chance of recurrence. Such complications can be avoided by generous calcaneocuboid resection that allows full correction and diminishes medial soft tissue tension.

Furthermore, the limitations of isolated talectomy in the correction of residual midfoot and forefoot deformities at the time of surgery have been studied [22]. Lateral column procedures, including resection of the anterior end of the calcaneus, calcaneocuboid joint resection with fusion, cuboid decancellation, and calcaneocuboid fusion have been used in tandem with talectomy for midfoot correction [10]. In the group B patients, these procedures were used more frequently than in group A. This could explain the high recurrence rate, and the requirement for further surgeries in group A.

One of the main limitations of this retrospective study was the small sample size. With that in mind, this paper provides a descriptive report of our patient population without statistical comparisons, as our samples would not be powered for such analyses. Thus, expanding the study population to different medical centers in Saudi Arabia and possibly to different Arabian Gulf countries in future studies should be considered. Furthermore, functional outcomes and patient satisfaction were not analyzed. The historical nature of the comparison group in our study may have resulted in bias. Also, we present the complications and differences in surgical techniques used for talectomy in a mixed group of patients with no consideration for the primary diagnosis or the nature of the procedure (primary or salvage). Despite these limitations, our findings suggest complications are associated with talectomy.

## Conclusions

Talectomy is an effective procedure for the treatment of persistent foot deformities, despite associated complications. Attention to detail during surgery, and addressing associated deformities with adjunct surgical interventions, should be considered.

## Data Availability

The datasets generated during and analyzed during the current study are not publicly available due to our hospital/national guidelines but are available from the corresponding author on reasonable request.

## Abbreviations

KFSH&RC: King Faisal Specialist Hospital & Research Centre
CP: Cerebral palsy
